# Lack of MEF2A Δ7aa mutation in Irish families with early onset ischaemic heart disease, a family based study

**DOI:** 10.1186/1471-2350-7-65

**Published:** 2006-07-27

**Authors:** Paul G Horan, Adrian R Allen, Anne E Hughes, Chris C Patterson, Mark Spence, Paul G McGlinchey, Christine Belton, Tracy CL Jardine, Pascal P McKeown

**Affiliations:** 1 Regional Medical Cardiology Centre, Royal Victoria Hospital, Grosvenor Road, Belfast, BT12 6BA, Northern Ireland, UK; 2 Department of Medicine, Queen's University Belfast, Institute of Clinical Science, Grosvenor Road, Belfast, BT12 6BJ, Northern Ireland, UK; 3 Department of Medical Genetics, Queen's University Belfast, Institute of Pathology, Grosvenor Road, Belfast, BT12 6BJ, Northern Ireland, UK; 4 Department of Epidemiology and Public Health, Queen's University Belfast, Mulhouse Building, Grosvenor Road, Belfast, BT12 6BJ, Northern Ireland, UK

## Abstract

**Background:**

Ischaemic heart disease (IHD) is a complex disease due to the combination of environmental and genetic factors. Mutations in the MEF2A gene have recently been reported in patients with IHD. In particular, a 21 base pair deletion (Δ7aa) in the MEF2A gene was identified in a family with an autosomal dominant pattern of inheritance of IHD. We investigated this region of the MEF2A gene using an Irish family-based study, where affected individuals had early-onset IHD.

**Methods:**

A total of 1494 individuals from 580 families were included (800 discordant sib-pairs and 64 parent-child trios). The Δ7aa region of the MEF2A gene was investigated based on amplicon size.

**Results:**

The Δ7aa mutation was not detected in any individual. Variation in the number of CAG (glutamate) and CCG (proline) residues was detected in a nearby region. However, this was not found to be associated with IHD.

**Conclusion:**

The Δ7aa mutation was not detected in any individual within the study population and is unlikely to play a significant role in the development of IHD in Ireland. Using family-based tests of association the number of tri-nucleotide repeats in a nearby region of the MEF2A gene was not associated with IHD in our study group.

## Background

The search for novel polymorphisms that increase the likelihood of IHD has recently led to the discovery of a potentially important role of the myocyte enhancing factor 2A gene (MEF2A). In 2003, Wang and colleagues [1] reported that they had identified a 21 base pair (bp) deletion (Δ7aa) of the MEF2A gene in a family with IHD, where the disease appeared to have an autosomal dominant pattern of inheritance. Subsequent investigation by the same research group revealed 3 further mutations in a separate part of the MEF2A gene. These authors suggest that mutations in the MEF2A gene may play a substantial role in the development of IHD.

Attempts to confirm this association to date have not been successful in case-control studies in both a Canadian [3] and, more recently, a Japanese population [4]. We sought to investigate the role of the Δ7aa mutation in a large family based study of patients with early-onset IHD in Ireland.

## Methods

### Subjects

Recruitment of the study population took place between August 1999 and October 2004. The inclusion criteria are described in detail in a previous publication [5]. Briefly, individuals were Caucasian with all four grandparents born in Ireland. Each family was required to have at least one family member affected with proven premature IHD (disease onset ≤ 55 years for males and ≤ 60 years for females) and at least one unaffected sibling and/or both parents surviving. Proven IHD was defined by one or more of the following: previous myocardial infarction (MI), previous unstable angina (typical chest pain with dynamic ECG changes or minor elevation of cardiac markers) or stable angina with angiographic evidence of obstructive coronary disease (>70% stenosis). Unaffected siblings were required to be 3 years older than the affected sibling at age of diagnosis of IHD and have no evidence of previous IHD using the "Rose chest pain on effort and possible infarction questionnaire" [6] and a standard 12 lead electrocardiogram independently coded using the "Minnesota code" [7].

Written informed consent was obtained from each patient. The study was approved by the Research Ethics Committee of Queen's University Belfast and the investigation conforms to the principles outlined in the Declaration of Helsinki.

### Statistical analysis

Two family based tests of association were used to analyse the data: the combined transmission disequilibrium test (TDT)/sib-TDT and the pedigree disequilibrium test (PDT) [8,9]. These tests avoid the problem of population stratification that is found in case-control studies.

### Genotyping

Polymerase chain reaction (PCR) amplification of the Δ7aa region was undertaken, using a fluorescently labelled primer (MWG Biotech Ebersberg, Germany). The PCR conditions were as previously described [5]. The forward primer sequence was GCATCAAGTCCGAACCGATT and the reverse primer sequence was GGAGCGACCCATTTCCTGTC. Amplification products were run on a capillary ABI PRISM^® ^3100 Genetic Analyser with a commercially available size standard (ROX 400™, Applied Biosystems). Sequencing of a random sample of 10 individuals was performed on a capillary ABI PRISM^® ^3100 Genetic Analyser. Results were analysed by Sequencher™ (Gene Codes Corporation, Michigan, USA).

## Results

The risk factors for both probands and siblings are shown below in Table 1. Of note, there are more male probands, and more female siblings, this reflects the earlier onset of IHD in men compared with women. Smoking and diabetes are more common in the probands. However, hypertension and elevated levels of lipoproteins were less common in the probands probably reflecting the use of vasoactive drugs to reduce blood pressure and the widespread use of lipid lowering agents in this population.

**Table 1:** Risk factors in probands and their siblings with premature onset IHD.

**Risk factor**	**Probands**	**Siblings**
Age	52.0	56.0
Female	113 (19.5%)	429 (54.6%)
Male	467 (80.5%)	357 (45.4%)
Body mass index	28.5	28.2
Non smoker	116 (20.0%)	328 (41.7%)
Ex smoker (≥ 1 year)	249 (42.9%)	224 (28.5%)
Current smoker	215 (37.1%)	235 (29.9%)
Hypertension treatment	148 (25.5%)	177 (22.5%)
Systolic BP ≥ 140 mmHg	30 (5.2%)	239 (30.4%)
Diastolic BP ≥ 95 mmHg	1 (0.2%)	2 (0.3%)
Total hypertension	179 (30.9%)	418 (53.2%)
Known diabetes	53 (9.1%)	43 (5.5%)
Random blood sugar ≥ 11.1 mmol/l	2 (0.3%)	6 (0.7%)
Total cholesterol (mmol/l)	4.9	5.8
Low density lipoprotein (mmol/l)	2.9	3.4
Triglycerides (mmol/l)	2.4	2.3
High density lipoprotein (mmol/l)	1.3	1.2

A total of 1494 individuals from 580 families were included (803 discordant sib-pairs and 64 parent-child trios). Due to incomplete genotyping 13 individuals from 7 families were removed, leaving 1481 individuals from 573 families. Analysis was based on amplicon size of the Δ7aa region of the MEF2A gene.

The Δ7aa mutation was not found in any individual in the study sample. Differences in amplicon size were identified and were due to variation in the numbers of triplet repeats in a nearby region coding for glutamate or proline residues, as previously reported [3]. Using the TDT/sib-TDT and PDT (352 informative families), the number of triplet repeats was not found to be associated with disease in our study group (Table 2).

**Table 2:** Fragment size and association with IHD using the TDT.

**Fragment size**	**TDT**		**Sib-TDT**		**Combined TDT/sib-TDT**		**p value**
	
	Observed	Expected	Observed	Expected	Observed	Expected	
138	0	1	2	1.8	2	2.3	n/a
141	0	0	1	1.0	1	1.0	n/a
144	0	0	0	1.0	0	1.0	n/a
147	29	29	209	209.7	238	238.7	0.98
150	25	17	133	132.5	158	158.5	0.59
153	29	36	287	186.9	316	319.4	0.75
156	1	0	3	2.0	4	2.5	n/a
159	0	1	1	1.0	1	1.5	n/a

## Discussion

Although the majority of researchers working in the field of complex traits propose a common-variant, small-effect model, the possibility also exists of a rare- variant, large-effect model. Wang and colleagues [1] reported the Δ7aa mutation in a family of 13 patients who exhibited an autosomal dominant inheritance pattern of IHD. Subsequent work by the same group [2] was undertaken in 207 unrelated patients, with a diagnosis of IHD based on angiography or development of MI, and 191 control subjects. Three novel mutations in exon 7 were found in four patients and in none of their control subjects. However, other groups have not confirmed this work. Weng and co-workers [3] did identify the Δ7aa mutation in 3 individuals without evidence of IHD and further work within their families did not show any evidence for cosegregation of the mutation with early onset IHD. Similarly, Kajimoto and colleagues, in a Japanese population, screened the MEF2A gene in 379 patients with MI and 589 control individuals. They identified one nonsense mutation (R447X) but were uncertain regarding the significance of this finding, as the patient was elderly and had other conventional risk factors for ischaemic heart disease. Research performed by other groups have also identified similar variations in the number of glutamate residues, they to have not found this to be associated with IHD; however, other mutations may be [10].

## Conclusion

Our research suggests that the Δ7aa mutation of the MEF2A gene is unlikely to play a significant role in the development of IHD in the Irish population. In addition, statistical analysis using family-based methods suggests that the triplet repeat polymorphism in this gene is not associated with IHD in our study group.

## Abbreviations

Ischaemic heart disease: IHD

Myocardial infarction: MI

Transmission disequilibrium test: TDT

Pedigree disequilibrium test: PDT

Polymerase chain reaction: PDT

## Competing interests

The author(s) declare that they have no competing interests.

## Authors' contributions

Paul G Horan: Recruited subjects and processed samples, laboratory analysis and wrote the paper.

Adrian R Allen: designed laboratory processes and results collection.

Anne E Hughes: Supervised all laboratory procedures.

Chris C Patterson: Provided statistical analysis.

Mark S Spence: Recruited subjects and processed samples form 1999–2001.

Paul G McGlinchey: Recruited subjects and processed samples from 2000–2002.

Christine Belton: Performed the majority of laboratory work.

Tracy CL Jardine: Recruited subjects 2002–2004.

Pascal P McKeown: Supervising consultant 1999–2004.

## Pre-publication history

The pre-publication history for this paper can be accessed here:


